# Foot–ground interaction and clubhead speed: impulse-based energy transfer as the key mechanism in the golf swing

**DOI:** 10.3389/fspor.2026.1790645

**Published:** 2026-04-13

**Authors:** Pornthep Rachnavy, Khemchat Chaemklan, Dipak Kumar Agrawal, Soodkhet Pojprapai, Hathairat Rachnavy, Thanomsak Senakam

**Affiliations:** 1 School of Sports Science, Institute of Science, Suranaree University of Technology, Nakhon Ratchasima, Thailand; 2 Department of Interdisciplinary Science and Internationalization, Institute of Science, Suranaree University of Technology, Nakhon Ratchasima, Thailand; 3 School of Telecommunication Engineering, Faculty of Engineering, Suranaree University of Technology, Nakhon Ratchasima, Thailand; 4 School of Ceramic Engineering, Institute of Engineering, Suranaree University of Technology, Nakhon Ratchasima, Thailand; 5 Sports and Health Center, Suranaree University of Technology, Nakhon Ratchasima, Thailand; 6 Department of Sports Science, Faculty of Physical Education, Sports, and Health, Srinakharinwirot University, Ongkharak Campus, Nakhon Nayok, Thailand

**Keywords:** clubhead speed, energy flow, foot–ground interaction, golf biomechanics, kinetic chain, mediation analysis

## Abstract

**Introduction:**

Clubhead speed is a primary determinant of golf performance, typically linked to the kinetic chain. Although foot–ground interaction variables such as plantar pressure and center-of-pressure behavior associate with performance, the mechanisms linking these factors to clubhead speed remain unclear. This study investigated whether foot–ground interaction influences clubhead speed directly or through intermediate mechanisms, such as segmental sequencing and energy transfer.

**Methods:**

Thirty right-handed golfers (15 professionals, 15 high-level amateurs) performed maximal swings using a driver and a 7-iron. Data from both club types were pooled to identify generalized biomechanical mechanisms across equipment. Kinematics, ground reaction forces, and plantar pressure were recorded. Impulse-based energy transfer efficiency—defined as the effectiveness of mechanical impulse transmission from proximal segments to the club head—was computed. Principal component analysis-derived constructs for hierarchical regression and serial mediation models, controlling for skill level.

**Results:**

Foot–ground interaction variables explained 35.5% of the variance in clubhead speed. Adding trunk sequencing and impulse-based energy transfer efficiency increased the explained variance to 75.4% (adjusted $R^{2}=0.715$). Mediation analysis revealed that early-phase center-of-pressure behavior did not directly influence clubhead speed. Instead, a significant indirect pathway through impulse-based energy transfer efficiency was found (accounting for 55% of the total variance in the mediation model); trunk sequencing did not independently mediate this relationship, while skill level remained a significant covariate.

**Conclusion:**

Foot–ground interaction enhances clubhead speed primarily by facilitating efficient impulse-based energy transfer along the kinetic chain rather than through direct mechanical effects. These findings support integrating foot-pressure and energy-flow analyses for performance assessment and evidence-based training.

## 1 Introduction

Clubhead speed is widely recognized as the primary predictor of driving distance and overall performance in golf, showing a strong association with scoring potential across all competitive levels (1–3). While extensive biomechanical research consistently highlights that higher clubhead speed (CHS) is underpinned by coordinated whole-body motion and efficient energy transfer within the kinetic chain—the coordinated sequence of segments that transfers energy and momentum from the ground to the distal clubhead (4–6), the precise mechanistic pathways by which foundational lower-limb dynamics translate into maximal clubhead velocity have not been fully characterized (1, 7, 8).

The foot–ground interaction represents the initial mechanical interface of the kinetic chain and is pivotal in generating and directing forces during the golf swing (7, 9, 10). Ground reaction forces, plantar pressure distribution, and center-of-pressure (COP) displacement collectively reflect how golfers interact with the ground to initiate and regulate movement (11–13). Prior studies have demonstrated that skilled golfers exhibit distinct patterns of COP progression and plantar pressure loading compared with less skilled players, particularly during key swing phases (14, 15). While these characteristics are essential for effective weight transfer and impulse generation, the downstream biomechanical mechanisms linking these lower-limb inputs to ultimate clubhead speed—beyond simple correlation—require further investigation.

Beyond force generation, the temporal organization of segmental motion is a significant factor in effective energy transmission. Proximal-to-distal sequencing, characterized by sequential peaks in angular velocity progressing from the lower extremities through the trunk to the upper limbs, is a well-established hallmark of skilled golf swings (4, 6, 16, 17). This proximal-to-distal sequencing, specifically the temporal coordination between the pelvis and thorax (trunk sequencing), is essential for efficient energy transmission. The disruptions in this sequencing can attenuate energy flow and reduce performance, even in the presence of high force output (8, 18, 19). Despite its recognized importance, existing studies often examine kinematic sequencing and ground reaction force characteristics in isolation (5, 9, 20), limiting insight into how these elements interact dynamically across the kinetic chain.

Mechanical energy flow analysis provides an informative framework for quantifying intersegmental energy generation, absorption, and transmission (21–23). By integrating joint power and segmental power components, energy flow metrics provide direct insight into the efficiency of intersegmental coordination during dynamic movements such as the golf swing (4, 17, 24). In particular, the concept of impulse-based energy transfer provides a mechanistic lens through which the foundational forces generated at the lower limbs can be converted and transmitted as kinetic energy to the distal segments. This framework specifically quantifies the proportion of the generated linear and angular impulses that are effectively transmitted through the kinetic chain to maximize the clubhead’s final kinetic energy. Recent studies have highlighted the importance of trunk and hip energy transfer in high-speed striking motions (4, 6, 8, 25). However, the specific role of lower-limb mechanics in shaping clubhead speed through their influence on energy flow, as well as its coordination with trunk sequencing, has received limited empirical attention.

Therefore, the purpose of this study was to examine the mechanistic pathways by which foot–ground interaction is associated with clubhead speed, either directly or indirectly, through trunk sequencing and impulse-based energy transfer within a proximal-to-distal kinetic chain framework. To address this, hierarchical regression and serial mediation analyses were employed to quantify both direct and indirect biomechanical pathways, integrating plantar pressure, COP dynamics, segmental sequencing, and energy-flow measures within a unified analytical model. A serial mediation framework was prioritized over structural equation modeling to specifically test the hypothesized sequential, step-by-step transmission of energy along the kinetic chain while maintaining statistical robustness for the current sample size (*N* = 30). This integrated approach aims to bridge the gap between foundational force dynamics and ultimate distal performance in the golf swing.

## 2 Materials and methods

### 2.1 Study design and participants

#### 2.1.1 Sample size determination

Prior to data collection, an a priori sample size determination was conducted using G*Power software (26). The analysis was based on an *F*-test for the overall multiple linear regression model (fixed effects, $R^{2}$ deviation from zero). A large expected effect size ($f^{2}$ = 0.35), a significance level of α=0.05, and a target statistical power of 0.80 were assumed. Under these conditions, the analysis indicated that a minimum of 23 participants was required for a model including five predictor variables.

#### 2.1.2 Participants

Thirty right-handed golfers (15 professional and 15 amateur) were recruited for this study. The participants had a mean age of 24.20 ± 4.74 years, a mean body mass of 75.80 ± 20.00 kg, a mean height of 170.60 ± 5.41 cm, and a mean body mass index of 25.83 ± 5.96 $\mathrm{kg}/m^{2}$. Participants were classified into two skill-level groups: professional golfers (certified members of the Thailand Professional Golf Association [TPGA], all of whom were actively competing at a national level or on a professional tour within the two years preceding the study) and amateur golfers (handicap ≤ 15; Mean handicap = 13.0 ± 2.9, range: 9–15).

A balanced representation of both sexes, consisting of 15 males and 15 females, was included (Professional: 7 males, 8 females; Amateur: 8 males, 7 females). This handicap threshold (≤15) was chosen to ensure that the amateur group represented “skilled” players with consistent swing mechanics, as golfers within this range typically exhibit a repeatable kinetic chain compared to higher-handicap recreational players. Because sex was not a primary analytical factor and preliminary screening revealed no systematic sex-related differences in the primary outcome variables, anthropometric characteristics were summarized descriptively at the group level.

Inclusion criteria required participants to be right-hand dominant, as confirmed by self-report and preferred golf swing hand, and to have no history of lower-limb or spinal musculoskeletal injury within the six months preceding the study. Exclusion criteria included left-hand dominance or the presence of any such injuries during the same period.

#### 2.1.3 Ethical approval

This study was approved by the Suranaree University of Technology Ethics Committee (EC-67-152). Prior to participation, all participants provided written informed consent.

### 2.2 Data acquisition

**Figure 1:**
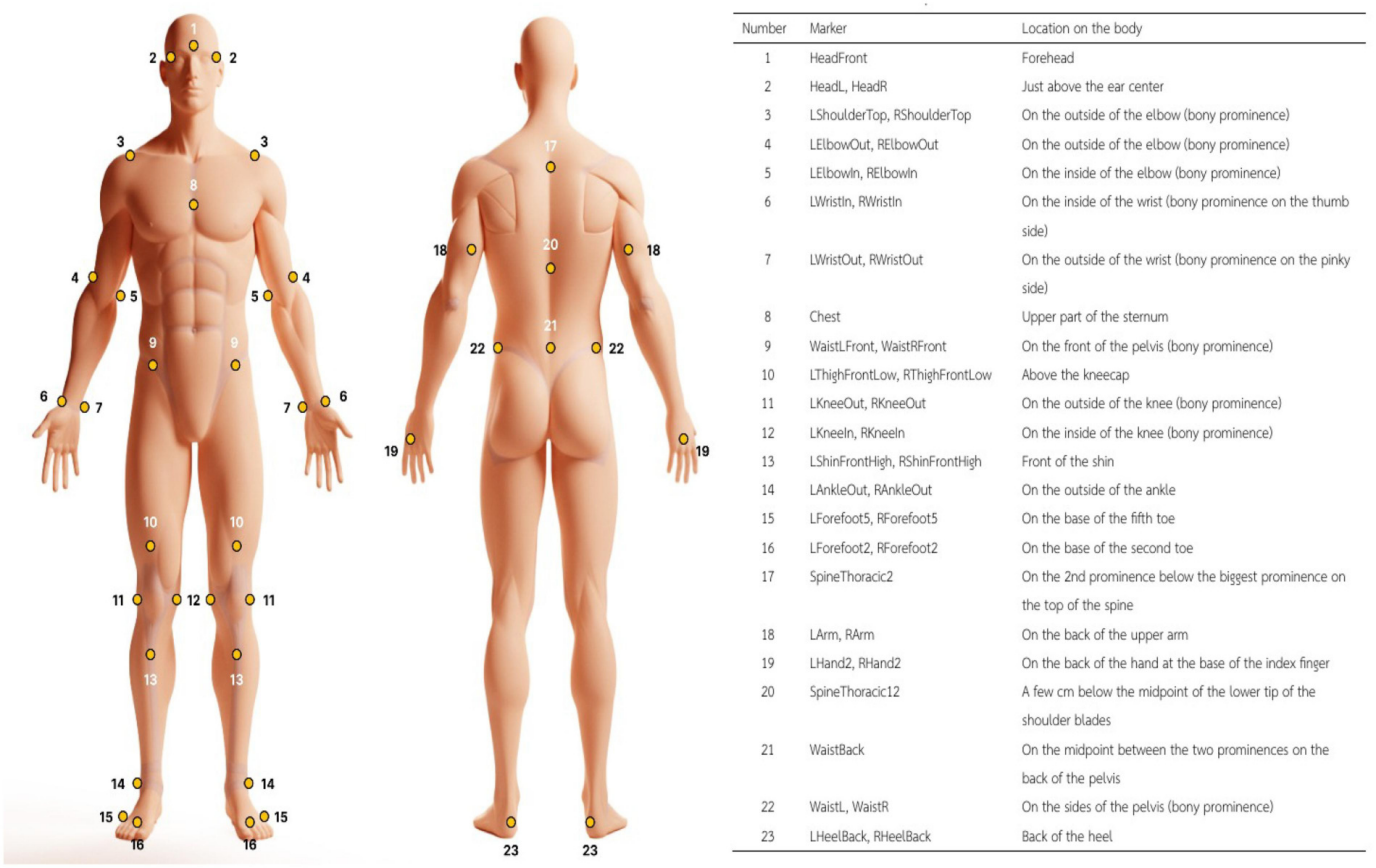
Reflective marker configuration for golf swing kinematics: 42 markers positioned on anatomical landmarks of the body and golf club, adhering to the Qualisys Sports Marker Set.

Synchronous 3D kinematic data, ground reaction forces, and plantar pressure data were collected while each participant performed five maximal-effort driver swings and five maximal-effort 7-iron swings. The analysis encompassed key biomechanical phases of the golf swing, with particular emphasis on the period surrounding ball impact, where peak force generation, mechanical impulse, and energy transfer occur. A total of forty-two retroreflective markers were affixed to designated anatomical landmarks on the participants and the club, following the Qualisys Sports Marker Set, as illustrated in Figure 1.^1^

**Figure 2:**
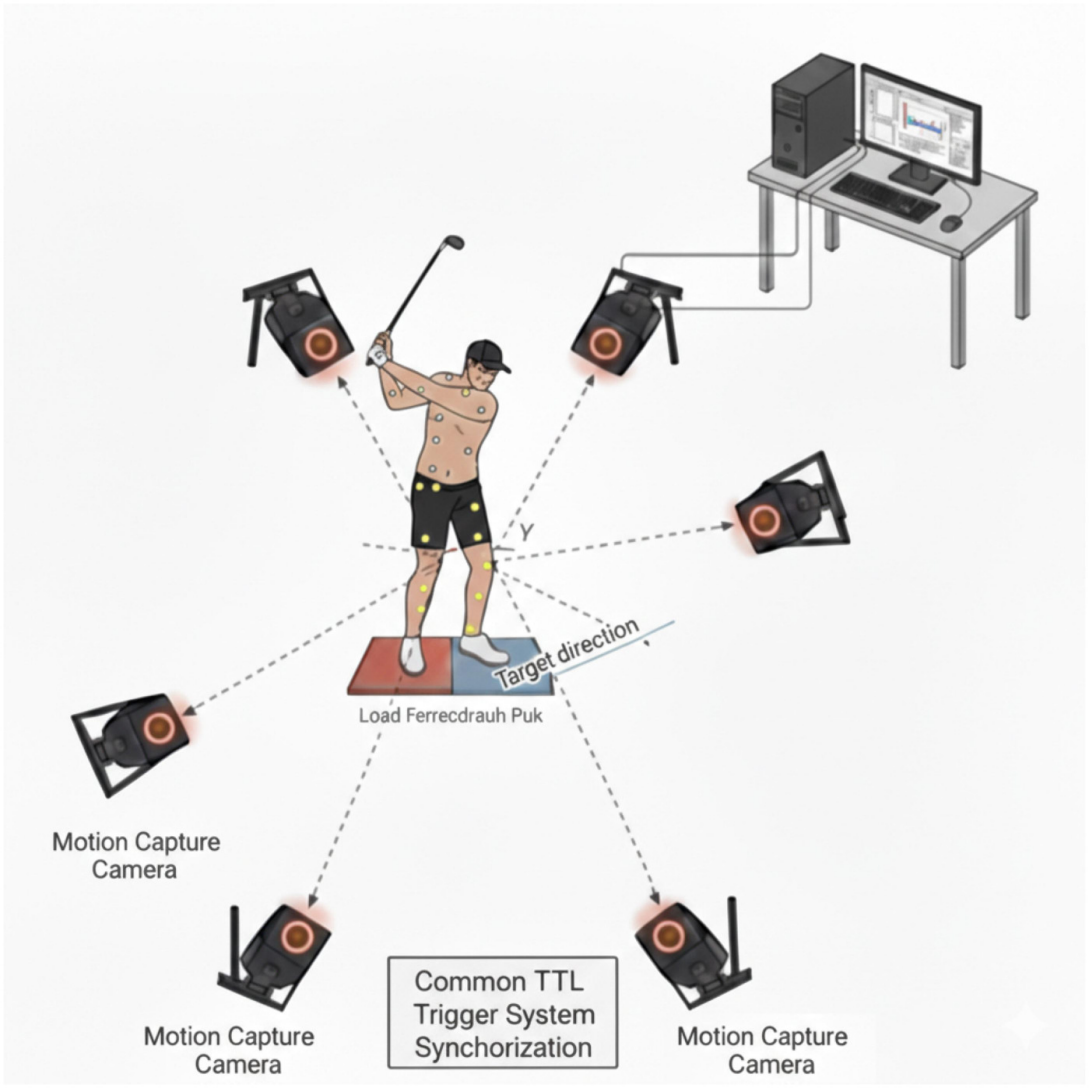
Experimental setup and TTL synchronization across systems.

**Figure 3:**
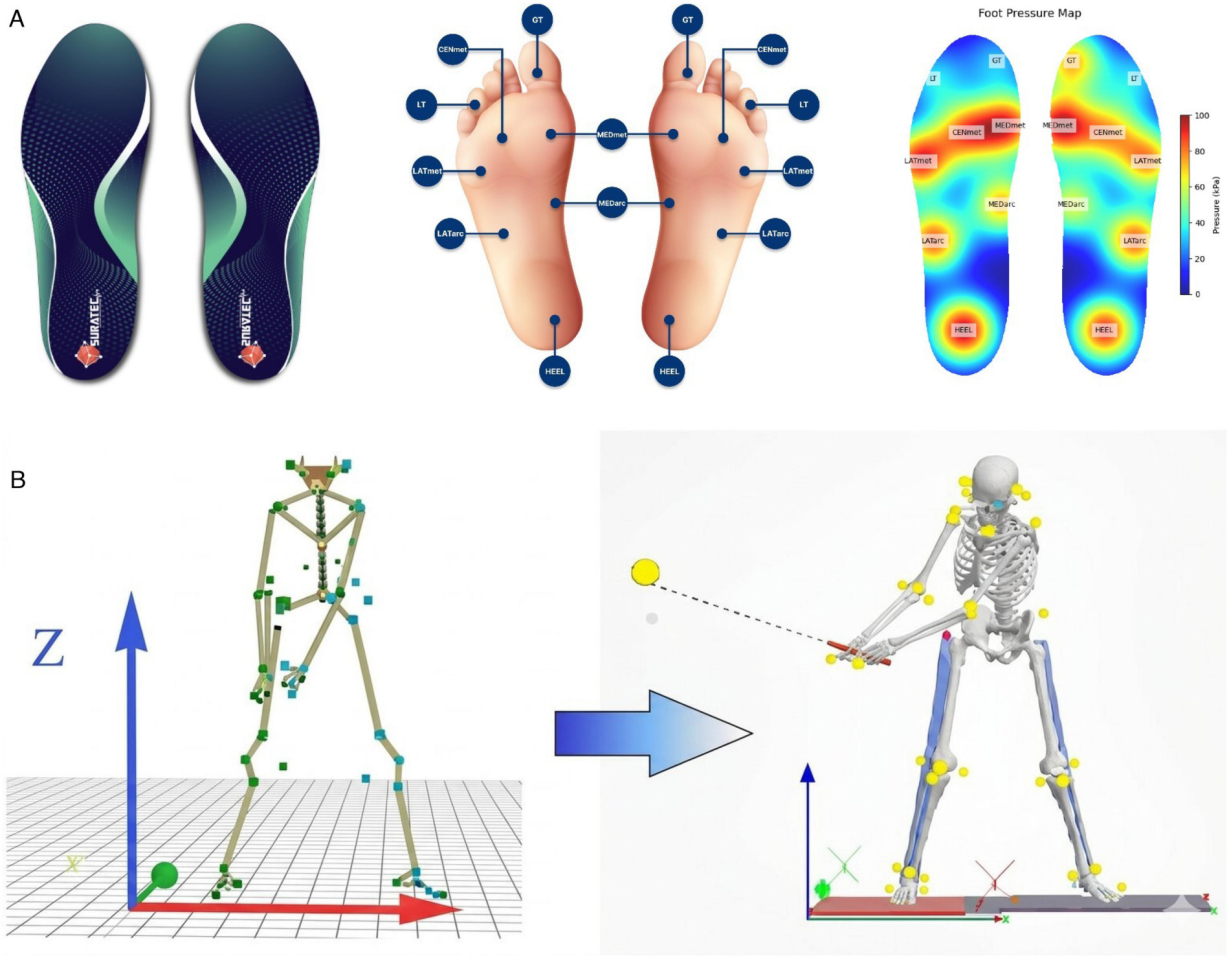
Experimental setup for foot–ground interaction and kinematic measurements. **(A)** Instrumented insole system and the definition of the eight plantar regions used for pressure distribution analysis: hallux, other toes, medial forefoot, central forefoot, lateral forefoot, medial midfoot, lateral midfoot, and heel. **(B)** Force plate measurement system showing the alignment with the global laboratory coordinate system used to quantify ground reaction forces and center-of-pressure trajectories during the golf swing.

Kinematic data were recorded using six optoelectronic cameras (Oqus 7+ series, Qualisys AB, Sweden) at a sampling frequency of 200 Hz (Figure 2). Ground reaction forces were measured separately for each foot using a pair of force platforms (Kistler 9286BA, Kistler Group, Switzerland) sampled at 1,500 Hz. Plantar pressure distribution was recorded using instrumented insole sensors (Surasole Pro 8, Suratec Co., Ltd., Nakhon Ratchasima, Thailand) at a sampling rate of 20 Hz (Figure 3A). While this sampling rate is lower than the kinematic and force platform frequencies, it was deemed sufficient for the objectives of this study, which focused on phase-integrated impulse and phase-averaged center of pressure variables rather than high-frequency instantaneous transients. Given that the downswing duration typically spans 0.2–0.3 s, the 20 Hz frequency provides adequate data points to capture robust biomechanical trends across the defined swing phases, as the integration process used to calculate pressure-impulse mitigates the impact of lower temporal resolution. Each insole incorporated eight resistive sensors strategically positioned to capture pressure variations across predefined foot regions. The Surasole Pro 8 system has been previously validated against gold-standard measurement systems, demonstrating high agreement with Kistler force plates (Bland-Altman Index = 93.68%) and exhibiting high reliability (Cronbach’s α = 0.92) during dynamic activities (27–29). These validations were conducted under ISO/IEC 17025 standards, ensuring its accuracy in capturing plantar pressure distribution and center-of-pressure trajectories (29).

During post-processing, plantar pressure data were segmented into eight anatomical foot areas. The sensors are interfaced with a microcontroller via a voltage divider circuit, and the outputs are digitized using a 10-bit analog-to-digital converter. Each sensor was individually calibrated to operate within a full-scale force range of 0–20 kg per sensor and exhibited a response time of less than 10 μs. Kinematic reconstruction and joint coordinate definitions were based on a global laboratory coordinate system (Figure 3B). All measurement systems were temporally synchronized via a common TTL trigger (Figure 2).

### 2.3 Data processing

Raw kinematic data were low-pass filtered at 12 Hz, and ground reaction force data at 50 Hz, using a fourth-order Butterworth filter (22). The choice of these cutoff frequencies follows established methodological standards in golf biomechanics to ensure that the high-frequency transients of the downswing are preserved while effectively removing measurement noise, as confirmed by residual analysis of the signal-to-noise ratio in preliminary trials. Visual3D software (C-Motion, USA) was utilized to compute joint kinetics and kinematics using a 14-segment full-body model with 26 degrees of freedom. Clubhead speed was determined as the first derivative of the 3D position of the clubhead marker, with peak linear velocity at the instant of impact serving as the primary outcome measure. The peak linear velocity at the precise instant of ball impact was extracted and used as the primary outcome measure, representing the terminal result of the kinetic chain’s energy transfer. Mechanical impulse was determined by integrating the ground reaction force over specific time intervals. Center of pressure displacement and plantar pressure distribution was derived from the plantar pressure system data, segmented into eight-foot regions.

Joint-level energy flow and impulse-based energy transfer variables were calculated based on established methodologies from kinetic chain analysis (30–32), quantifying the transfer of mechanical energy between adjacent segments across all major joints within the 14-segment, 26-degree-of-freedom full-body model. Impulse-based energy transfer, crucial for understanding how foundational forces generated from foot-ground interaction are transmitted, was specifically quantified using these joint-level energy flow metrics. Specifically, segmental power analysis for the trunk segment was employed to quantify the energy flow (4, 6, 32, 33).

The joint force power, segment torque power, and total segment power were calculated using established biomechanical formulations (see Equations 1–3). JFP was defined as the net power transferred through a joint due to joint reaction forces and linear velocities of the adjacent segments, whereas STP represented the power generated by joint moments and segment angular velocity. Consequently, Total Segment Power represents the algebraic sum of JFP and STP: ?>

$$\mathrm{JFP}=F_{p}\cdot v_{p}-F_{d}\cdot v_{d}$$ (1)

$$\mathrm{STP}=M_{p}\cdot \omega -M_{d}\cdot \omega$$ (2)

SP=JFP+STP (3)

In these equations, F denotes the joint reaction force vector, v represents the linear velocity of the joint center, M corresponds to the joint moment vector, and ω indicates the angular velocity of the segment. The subscripts p and d refer to the proximal and distal joints of the segment, respectively. These computations were conducted to quantify the generation, absorption, and transmission of mechanical energy within the musculoskeletal system (6, 30, 31, 34). For energy flow analysis, a positive value was defined as energy generation or power inflow into the segment, while a negative value represented energy absorption or power outflow from the segment. Net energy transfer was computed by integrating the total segment power (SP) over the duration of each identified swing phase to derive the total mechanical work performed during that interval.

Energy transfer was quantified by calculating the net energy transfer rate, specifically the inflow or outflow of energy within a body segment, which was determined from the combined total of joint force power and segment torque power (22, 35, 36). For time-series-derived variables, values were averaged across five valid swings for each participant to improve measurement reliability and reduce within-subject variability. All plantar-pressure—derived and COP variables were computed as phase-averaged or phase-integrated measures corresponding to the predefined swing phases rather than instantaneous peak values.

### 2.4 Biomechanical phase identification

**Figure 4:**
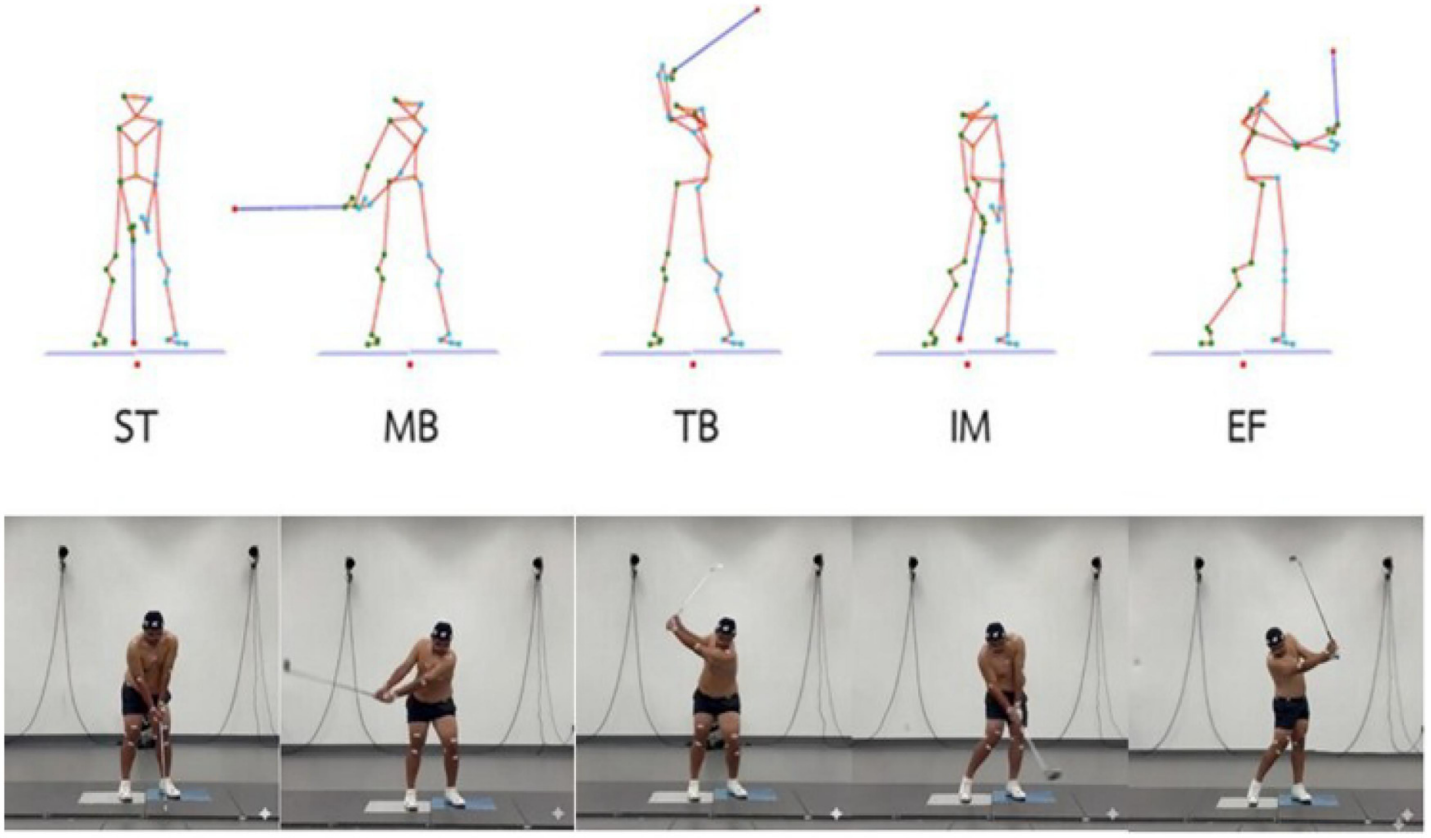
Phases of the golf swing analyzed in this study: Start (ST), Middle Backswing (MB), Top of Backswing (TB), Ball Impact (IM), and Early Finish (EF).

Objective definitions for golf swing events and phases were established using kinematic data, with the analyzed phases illustrated in Figure 4. The specific criteria for identifying each phase were as follows:

- **Start:** Characterized as the precise moment the clubhead begins its motion away from the ball (37, 38), often identified by a threshold in vertical clubhead speed after address (39) or when clubhead linear speed crosses $0.0\;m\;s^{-1}$ (40).
- **Middle backswing:** Defined as the instant the club shaft achieves a parallel position relative to the ground during the backswing (19, 24).
- **Top of backswing:** Designated as the specific point when the club’s backward motion stops or reverses, typically when the club reaches its maximum height or where the clubhead’s linear velocity in the global z-axis reaches its lowest negative point (19, 37, 40).
- **Ball impact:** Defined as the precise moment the clubhead strikes the golf ball (37, 38), or the moment immediately preceding the onset of ball velocity increase (39, 40).
- **Early finish:** Defined as the moment the club shaft becomes horizontally aligned with the ground throughout the follow-through phase (39, 40).

**Figure 5:**
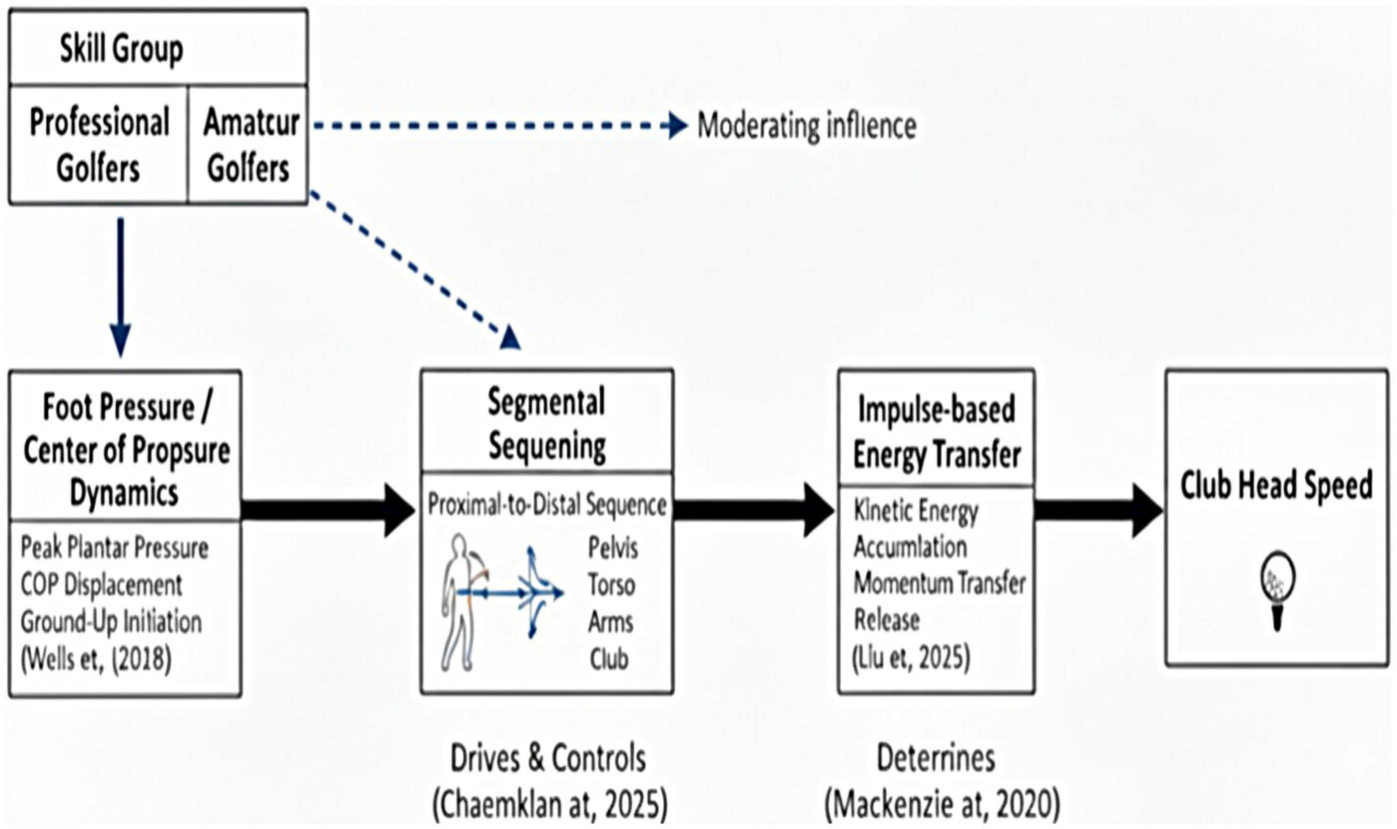
Hypothesized hierarchical kinetic chain model of golf swing performance: illustrating statistical associations and mediating roles of foot dynamics and energy transfer under the influence of skill level.

### 2.5 Statistical analysis

IBM SPSS Statistics (Version 29; IBM Corp., Armonk, NY, USA) was employed for all statistical analyses. Normally distributed variables are reported using means and standard deviations (M ± SD), while those not normally distributed are presented as medians and interquartile ranges. A significance level of α=0.05 was established as a priori for all statistical tests. Data from driver and 7-iron swings were pooled to identify a generalized kinetic chain mechanism. This was justified by a Principal Component Analysis which revealed a single-component solution accounting for 84.62% of the total variance, indicating structural similarity between the two club types.

#### 2.5.1 Data screening and assumption testing

Prior to inferential analysis, all variables were screened for missing values, univariate outliers, and distributional properties. Normality was assessed by Shapiro–Wilk tests, alongside a visual appraisal of Q–Q plots, and the examination of skewness–kurtosis coefficients (41, 42). Multicollinearity among predictor variables was evaluated using variance inflation factors, with values <5.0 indicating acceptable levels. Additionally, bivariate correlations (*r*) between factor scores from different blocks were examined to ensure they did not exceed the 0.85 threshold, thereby confirming the statistical independence and stability of the predictors used in the final models.

##### 2.5.1.1 Group comparisons

To compare biomechanical variables between Professional and amateur golfers (with handicaps no greater than 15), independent samples *t*-tests were utilized. In instances where normality assumptions were violated, Mann–Whitney *U* tests were employed. Effect sizes were computed utilizing Cohen’s d, with their interpretation guided by the conventional thresholds for small (0.20), medium (0.50), and large (0.80) (43).

##### 2.5.1.2 Correlation analysis

To evaluate bivariate relationships among plantar pressure variables, center of pressure metrics, impulse measures, energy flow variables, joint sequencing parameters, and clubhead speed Pearson’s product-moment correlation coefficients (*r*) were determined.

##### 2.5.1.3 Principal component analysis

To reduce the dimensionality of the extensive biomechanical dataset and identify underlying patterns of movement, an exploratory principal component analysis (PCA) was conducted using the correlation matrix to account for the differing scales and units among kinematic and kinetic variables. This dataset comprised approximately 500 biomechanical variables, including joint-specific energy flow metrics of the ankle, knee, hip, trunk, shoulder, elbow, and wrist (quantified bilaterally for left and right sides across five swing phases: stance, mid-backswing, top of backswing, impact, and early follow-through). While the primary focus of this study is the lower-limb-to-trunk pathway, these distal upper-limb metrics were included in the initial PCA to ensure a comprehensive representation of the entire kinetic chain and to capture the total variance in energy transmission. This holistic approach allowed for the identification of primary variance-explaining components before isolating the specific lower-limb and trunk dynamics for the subsequent mediation analysis. Regional plantar pressure distributions from eight foot regions per side; center-of-pressure trajectories in the mediolateral and anteroposterior directions; segmental sequencing parameters along the kinetic chain; and inter-phase mechanical impulse transfer indices between successive swing phases. Clubhead speed was treated as a separate performance outcome measure and was not included among the PCA input variables. To manage this high-dimensional dataset with a sample size of N=30, a block-wise PCA approach was employed where variables were partitioned into four conceptually distinct functional domains: Joint energy flow (304 variables, 10.1:1 variable-to-participant ratio), Segment sequencing (136 variables, 4.5:1 ratio), COP/plantar pressure (40 variables, 1.3:1 ratio), and Impulse metrics (20 variables, 0.7:1 ratio). While the participant-to-variable ratio in certain blocks is lower than traditional recommendations for factor discovery, this block-wise approach was utilized strictly as a dimensionality reduction technique to identify the primary variance-explaining components for subsequent mediation modeling, thereby minimizing collinearity while ensuring biomechanical interpretability.

Prior to factor extraction, the appropriateness of the data was confirmed using the Kaiser–Meyer–Olkin measure, with all values exceeding 0.75, and Bartlett’s test of sphericity (p<0.001), supporting the suitability of the dataset for dimensionality reduction. Initially, a number of components equal to the total number of variables in each domain—304 for joint energy flow, 136 for segment sequencing, 40 for COP/plantar pressure, and 20 for impulse metrics—were extracted. Following this, factors were retained based on the Kaiser criterion (eigenvalues > 1.0) and visual inspection of the scree plots (see Supplementary Figure S1), collectively accounting for 71.6% of the total variance. A Varimax rotation was applied to enhance interpretability of the extracted components, ensuring that factors extracted within each functional domain were orthogonal and perfectly uncorrelated. The resulting principal components, representing distinct biomechanical movement patterns, were saved specifically as standardized regression factor scores and subsequently entered hierarchical multiple linear regression and mediation analyses. The resulting principal components, representing distinct biomechanical movement patterns, were saved as standardized regression scores and subsequently entered hierarchical multiple linear regression and mediation analyses.

##### 2.5.1.4 Hierarchical multiple linear regression

Hierarchical multiple linear regression analyses were performed to ascertain the predictors of clubhead speed. Predictor variables were entered in theoretically driven blocks reflecting the proximal-to-distal kinetic chain framework. The incremental variance explained was assessed at every stage. To ensure model stability and mitigate potential overfitting given the sample size (N=30), Adjusted R-squared values were prioritized as a conservative measure of explanatory power. Furthermore, consistent with the mediation approach, the stability of the regression estimates was supported by a bias-corrected bootstrap resampling procedure with 5,000 resamples to provide robust estimates and minimize sensitivity to the participant-to-predictor ratio. While the sample size (N=30) was determined for regression analysis, we acknowledge that it may be at the lower threshold for detecting smaller indirect effects in subsequent mediation analyses. To mitigate this, we prioritized bias-corrected bootstrapping to maintain statistical power and ensure the robustness of the reported effects.

##### 2.5.1.5 Serial mediation analysis

To examine the mechanistic pathways linking lower-limb mechanics to clubhead speed, mediation and serial mediation analyses were performed utilizing the PROCESS macro within SPSS (44). A bias-corrected bootstrap procedure, incorporating 5,000 resamples, was employed to estimate indirect effects. To enhance interpretability, the serial mediation model was specified using a system of three structural equations (see Equations 4–6), as illustrated in Supplementary Figure S2: ?>

$$M_{1}=i_{1}+a_{1}X+\sum \beta_{\mathrm{cov}}\cdot \mathrm{Covariates}+e_{1}$$ (4)

$$M_{2}=i_{2}+a_{2}X+d_{21}M_{1}+\sum \beta_{\mathrm{cov}}\cdot \mathrm{Covariates}+e_{2}$$ (5)

$$Y=i_{3}+c^{\prime }X+b_{1}M_{1}+b_{2}M_{2}+\sum \beta_{\mathrm{cov}}\cdot \mathrm{Covariates}+e_{3}$$ (6)

The reported serial mediation model included the Foot–Ground Interaction factor as the predictor (X), Trunk Kinematic Sequencing ($M_{1}$) and Impulse-based energy transfer efficiency ($M_{2}$) as sequential mediators, and clubhead speed as the outcome (Y), with skill group included as a covariate. This model configuration accounted for a substantial proportion of the variance in clubhead speed ($R^{2}=0.55$). To maintain reporting consistency across all analyses, 95% Confidence Intervals were provided for all direct effects and regression coefficients in Table 4, complementing the indirect effect CIs reported in Table 5.

#### 2.5.2 Effect size, model evaluation, and power

The final sample size was considered sufficient to detect medium-to-large effects with adequate statistical power, particularly when effect sizes are substantial and theoretical constraints guide the model (45). While the final sample size of N=30 is consistent with previous golf biomechanics research, we acknowledge that mediation analyses generally require larger samples to detect smaller indirect effects with high statistical power. However, based on the observed large effect size ($f^{2}=0.35$) for the primary mechanistic pathway involving impulse-based energy transfer efficiency, a post-hoc power calculation confirmed that the achieved power exceeded 0.80. This level of power provides confidence that the study was sufficiently equipped to identify the significant indirect effects reported, despite the inherent limitations of small-to-medium sample sizes in detecting subtler influences.

## 3 Results

### 3.1 Descriptive statistics and group comparisons

Descriptive statistics and between-group comparisons for participant characteristics, biomechanical factors, and performance outcomes are detailed in Table 1. Age did not significantly differ when comparing professional and amateur golfers (t=−0.85, p=0.424, Cohen’s d = −0.31), body mass (t = 0.86, p = 0.394, Cohen’s d = 0.32), height (t = 0.61, p = 0.551, Cohen’s d = 0.22), or body mass index (t = 0.73, p = 0.474, Cohen’s d = 0.27). All effect sizes for participant characteristics were small, indicating comparable anthropometric profiles between the two groups.

**Table 1:** Descriptive statistics and between-group comparisons of participant characteristics, biomechanical factors, and performance outcomes.

Variable	Professional	Amateur	Test	*p*-value	Effect size
	(N=15)	(N=15)	statistic		(d)
Participant characteristics					
Age (years)	24.27±4.47	25.47±3.20	−0.85	0.424	−0.31
Weight (kg)	75.00±20.00	70.00±10.06	0.86	0.394	0.32
Height (cm)	169.87±5.41	168.73±4.86	0.61	0.551	0.22
BMI (kg/$m^{2}$)	25.83±5.96	24.56±3.24	0.73	0.474	0.27
Sex (Male/Female)	7/8	8/7			
Biomechanical factors					
Early-phase COP displacement	0.10±1.10	−0.10±0.91	0.56	0.583	0.20
Late-phase COP stabilization	0.05±1.01	−0.05±1.02	0.26	0.793	0.10
Trunk sequencing at impact	−0.84±0.41	0.84±0.62	−8.73	<0.001	−3.19
Impulse-related energy transfer efficiency	−0.54±0.88	0.54±0.82	−3.46	0.002	−1.27
Performance outcome					

Data are presented as mean ± standard deviation. Test statistics represent *t*-values derived from independent-samples *t*-tests (two-tailed). Effect sizes are reported as Cohen’s d, calculated using pooled standard deviations.

With respect to biomechanical factors, statistically, no significant inter-group distinctions were identified concerning early-phase center-of-pressure displacement (t = 0.56, p = 0.583, Cohen’s d = 0.20) or late-phase COP stabilization (t = 0.26, p = .793, Cohen’s d = 0.10), indicating comparable COP control strategies across professional and amateur golfers during the swing’s initial and stabilization phases. In contrast, trunk sequencing at impact differed markedly between groups, with professional golfers exhibiting significantly lower trunk sequencing index values than amateur golfers (t=−8.73, p<0.001), accompanied by an extremely large effect size (Cohen’s d = −3.19). Because the variables loading on this factor represent the timing of peak trunk angular velocity and pelvis–trunk separation relative to impact, lower values of this index correspond to a more temporally coordinated proximal-to-distal sequencing pattern. This interpretation is consistent with the directional meaning of the PCA-derived trunk sequencing factor described in Section 3.2. Impulse-related energy transfer efficiency was significantly greater in the professional group (t=−3.46, p = 0.002), with a large effect size (Cohen’s d = −1.27), further supporting the notion that superior golf performance is associated with enhanced intersegmental coordination and more effective mechanical energy transfer at impact.

Regarding performance outcomes, clubhead speed was significantly higher in professional golfers (110.91 ± 4.12 mph) compared with amateur golfers (91.97 ± 4.07 mph), with a very large between-group difference (*t* = 12.67, p<0.001, Cohen’s d = 4.63). Collectively, these findings indicate that while professional and amateur golfers exhibit comparable demographic characteristics and similar COP control during early and late swing phases, substantial differences emerge in trunk sequencing, impulse-related energy transfer efficiency, and clubhead speed. These findings underscore the pivotal contribution of proximal–distal coordination and efficient energy transfer mechanisms in differentiating golf performance levels.

### 3.2 Dimensionality reduction and factor structure

To manage the high dimensionality of the biomechanical dataset, which comprised joint-specific mechanical energy flows, plantar pressure distributions, center-of-pressure trajectories, segmental sequencing variables, and impulse measures across multiple swing phases, a hierarchical, theory-driven dimensionality reduction strategy was adopted.

Principal component analysis, employing Varimax rotation, was performed separately within conceptually defined variable blocks: foot pressure and COP metrics, joint-level energy flow variables, segmental sequencing indices, and impulse-based measures. This rotation ensured that the extracted components within each conceptual block were orthogonal and perfectly uncorrelated (r = 0). The appropriateness of the sample was confirmed through the Kaiser–Meyer–Olkin measure (all KMO values > 0.75), and Bartlett’s test of sphericity (all p<0.001) supported the data’s suitability for factor extraction.

Across these blocks, a total of four latent factors were extracted adhering to the criterion of eigenvalues exceeding 1.0, which, in aggregate, explained approximately 71.6% of the total variance. The factor loadings for each extracted component are presented in Table 2, demonstrating consistent patterns with biomechanical expectations and clear simple structure.

**Table 2:** Factor loadings and explained variance for extracted biomechanical factors from the principal component analysis, with significant loadings (>0.70) shown in bold.

Variable	Early-phase COP displacement	Late-phase COP stabilization	Trunk sequencing at impact	Impulse-related energy transfer efficiency
COP anterior–posterior displacement (address → mid-backswing)	0.82	0.21	0.18	0.09
COP medial–lateral displacement (address → mid-backswing)	0.78	0.19	0.14	0.11
Peak lead-foot plantar pressure (early backswing)	0.74	0.26	0.12	0.17
COP variability (impact phase)	0.18	0.81	0.22	0.13
COP velocity at impact	0.24	0.77	0.19	0.16
Lead-foot plantar pressure stability index (late downswing)	0.21	0.73	0.17	0.25
Peak trunk angular velocity timing (relative to pelvis)	0.19	0.23	0.84	0.28
Pelvis–trunk separation angle at impact	0.22	0.18	0.81	0.31
Trunk angular velocity at impact	0.17	0.21	0.79	0.34
Hip-to-trunk energy flow (top of backswing → impact)	0.14	0.26	0.33	0.82
Trunk-to-arm energy flow (downswing)	0.11	0.24	0.29	0.79
Net mechanical impulse (lower limb → trunk)	0.16	0.28	0.31	0.76
**Explained variance (%)**	**21.4**	**18.9**	**16.7**	**14.6**
**Cumulative variance (%)**	**21.4**	**40.3**	**57.0**	**71.6**

The resulting latent factors represented distinct functional constructs:

- **Early-Phase COP Displacement:** Reflecting early-phase COP displacement patterns.
- **Late-Phase COP Stabilization:** Reflecting late-phase COP stabilization.
- **Trunk Sequencing at Impact:** Reflecting trunk sequencing characteristics near impact.
- **Impulse-Related Energy Transfer Efficiency:** Reflecting impulse-related energy transfer efficiency during the transition from top-backswing to impact.

It should be noted that the variables loading strongly on the Trunk Sequencing at Impact factor (e.g., peak trunk angular velocity timing and pelvis–trunk separation angle) represent event timing relative to impact. Consequently, higher factor scores reflect relatively delayed trunk rotational events, whereas lower scores indicate earlier trunk motion within the downswing and therefore more coordinated proximal–distal sequencing. Accordingly, lower scores on this factor correspond to the sequencing pattern more commonly observed in the professional group.

Standardized factor scores for each of these constructs were computed and retained for all subsequent analyses.

### 3.3 Inter-factor associations

Bivariate correlations among all retained factor scores were examined to evaluate factor independence and potential multicollinearity. The correlation matrix is presented in Table 3. Correlation coefficients ranged from moderate to moderately strong (*r* = 0.29–0.63), indicating meaningful associations among biomechanical constructs without excessive redundancy. No correlation exceeded the critical threshold of |r|>0.85, confirming that the extracted factors captured related yet distinct aspects of swing mechanics suitable for subsequent multiple regression and mediation analyses. Furthermore, the stability of these factors was confirmed by variance inflation factors (VIF<3.0), indicating no evidence of problematic multicollinearity.

**Table 3:** Inter-factor correlations among extracted biomechanical constructs.

Factor	1. Early-phase COP displacement	2. Late-phase COP stabilization	3. Trunk sequencing at impact	4. Impulse-related energy transfer efficiency
1. Early-phase COP displacement	1			
2. Late-phase COP stabilization	0.34${}^{*}$	1		
3. Trunk sequencing at impact	0.41${}^{*}$	0.29${}^{*}$	1	
4. Impulse-related energy transfer efficiency	0.38${}^{*}$	0.46${}^{*}$	0.63${}^{*}$	1

Values represent Pearson’s correlation coefficients between standardized factor scores. *p<0.05.

Notably, the Trunk Sequencing at Impact factor demonstrated moderate associations with Early-Phase COP Displacement (r=0.41,p<0.05) and a moderately strong association with Impulse-Related Energy Transfer Efficiency (r=0.63,p<0.01), suggesting a theoretically plausible interplay between trunk coordination at impact, postural control strategies, and mechanical energy transfer during the golf swing.

### 3.4 Determinants of clubhead speed

#### 3.4.1 Hierarchical multiple regression

A hierarchical multiple regression analysis was conducted to identify biomechanical determinants of clubhead speed (CHS). Factor scores were entered in a theory-driven sequence across three blocks, with Group (professional = 1, amateur = 0) included as a covariate in Block 1. The results of the hierarchical regression analysis are presented in Table 4.

- **Block 1:** Early-Phase COP Displacement, Late-Phase COP Stabilization, and Group membership collectively explained 35.5% of the variance in CHS $\left(R^{2}=0.355,\;\Delta R^{2}=0.355\right)$, F-change(3,26)=6.61, p=0.002. Neither Early-Phase COP Displacement (β=0.18,p=0.19) nor Late-Phase COP Stabilization (β=0.17,p=0.20) emerged as significant predictors. In contrast, Group membership was a significant predictor (β=0.71,p<0.001), indicating higher CHS among professional golfers.
- **Block 2:** The addition of Trunk Sequencing at Impact contributed an incremental 20.9% to the explained variance in CHS $\left(\Delta R^{2}=0.209\right)$, F-change =10.31, p=0.003. Trunk Sequencing at Impact was identified as a significant predictor of CHS (standardizedβ=0.39,p=0.003).
  Considering the direction of the factor score, lower scores on this factor correspond to a more coordinated proximal–distal trunk sequencing pattern, suggesting that swing patterns characterized by more efficient trunk coordination are associated with higher clubhead speed.
- **Block 3:** The inclusion of Impulse-Related Energy Transfer Efficiency further accounted for an additional 19.0% of the variance in CHS $\left(\Delta R^{2}=0.190\right)$, F-change =15.88, p<0.001. Impulse-Related Energy Transfer Efficiency emerged as a significant predictor (β=0.55,p<0.001).

**Table 4:** Hierarchical multiple regression analysis predicting clubhead speed from biomechanical factors.

Predictor	B	SE	β	*t*	95% CI	*p*
Block 1						
Early-phase COP displacement	0.72	0.54	0.18	1.33	−0.40 – 1.84	.190
Late-phase COP stabilization	0.65	0.49	0.17	1.32	−0.37 – 1.67	.200
Group (Professional = 1)	6.48	1.12	0.71	5.79	4.17 – 8.79	0.0001
Model summary						$R^{2}=.355$, $\Delta R^{2}=.355$ F change =6.61, p=.002
Block 2						
Trunk sequencing at impact	2.31	0.72	0.39	3.21	0.84 – 3.78	0.003
Model summary						$R^{2}=.564$, $\Delta R^{2}=.209$ F change =10.31, p=.003
Block 3						
Impulse-related energy transfer efficiency	3.84	0.67	0.55	5.73	2.46–5.22	0.0001
Final model summary						$R^{2}=.754$, Adjusted $R^{2}=.715$ F=19.17, p<.001

B, unstandardized regression coefficient; β, standardized regression coefficient; SE, standard error; CI, confidence interval; COP, center of pressure. Group was coded as professional = 1 and amateur = 0.

For the visual representation of these associations and the mediating role of impulse-related energy, see Supplementary Figure S2.

**Table 5:** Serial mediation analysis of early-phase COP displacement on clubhead speed.

Path	Effect (B)	SE	95% CI	*p*
Total effect				
Early-phase COP displacement → CHS	0.72	0.54	−0.40, 1.84	0.190
Direct effect				
Early-phase COP displacement → CHS (controlling for mediators)	0.21	0.47	−0.77, 1.19	0.660
Indirect effects				
Via trunk sequencing at impact ($M_{1}$)	0.06	0.09	−0.10, 0.24	0.480
Via impulse-related energy transfer efficiency ($M_{2}$)	0.31	0.14	0.07, 0.63	0.010
Serial mediation: $M_{1}\to M_{2}$	0.09	0.11	−0.08, 0.32	0.290

B, unstandardized regression coefficient; SE, standard error; CI, confidence interval; COP, center of pressure; CHS, clubhead speed.

Indirect effects were estimated using bias-corrected bootstrap confidence intervals based on 5,000 resamples. Confidence intervals represent 95% bootstrap confidence intervals. The mediation model explained a substantial proportion of the variance in clubhead speed ($R^{2}=.55$, F=7.60, p<.001).

In the final model, Impulse-Related Energy Transfer Efficiency (β=0.55,p<0.001), Trunk Sequencing at Impact (β=0.39,p=0.003), and Group membership (β=0.71,p<0.001) remained significant predictors of CHS. Early-Phase COP Displacement and Late-Phase COP Stabilization did not demonstrate significant direct associations with CHS in the final model (p>0.05). Overall, the final model explained 75.4% of the variance in CHS $\left(R^{2}=0.754,\;\text{adjusted}\;R^{2}=0.715\right)$, F=19.17, p<0.001, indicating excellent explanatory power.

Collinearity diagnostics indicated acceptable levels of multicollinearity (all VIFs <3). Residual analyses, including visual inspection of standardized residual plots and the Durbin–Watson statistic (1.98), revealed no major violations of normality, homoscedasticity, or independence of errors.

### 3.5 Serial mediation analysis: foot pressure → energy transfer → clubhead speed

Bootstrapped serial mediation analyses (5,000 resamples) were conducted to examine whether the relationship between Early-Phase COP Displacement and clubhead speed (CHS) was mediated by Trunk Sequencing at Impact (M1) and Impulse-Related Energy Transfer Efficiency (M2).

#### 3.5.1 Total and direct effects

The total effect of Early-Phase COP Displacement on CHS was not statistically significant (Total Effect = 0.72, SE = 0.54, p = 0.19). Similarly, the direct effect continued to be statistically non-significant after controlling for the mediating variables (Direct Effect = 0.21, SE = 0.47, p = 0.66), indicating that early COP behavior alone does not directly determine clubhead speed.

#### 3.5.2 Indirect effects

The indirect effect via Trunk Sequencing at Impact alone did not achieve statistical significance (Indirect Effect = 0.06, SE = 0.09, 95% CI [−0.10,0.24], p=0.48), suggesting that trunk sequencing did not independently mediate the relationship between early COP displacement and CHS.

In contrast, the indirect pathway through Impulse-Related Energy Transfer Efficiency achieved statistical significance (Indirect Effect = 0.31, SE = 0.14, 95% CI [0.07,0.63], p=0.01), identifying this factor as the primary mediating mechanism linking early COP behavior to clubhead speed.

The full serial mediation pathway (Early-Phase COP Displacement → Trunk Sequencing at Impact → Impulse-Related Energy Transfer Efficiency → CHS) did not achieve statistical significance (Indirect Effect = 0.09, SE = 0.11, 95% CI [−0.08,0.32], p=0.29). Notably, despite a significant moderately strong bivariate correlation between Trunk Sequencing and Impulse-Related Energy Transfer (r = 0.63, p<0.01; see Table 3), the non-significant serial indirect effect indicates that trunk sequencing did not function as a necessary intermediate step in the transmission of impulse-related energy.

#### 3.5.3 Mediator–outcome relationships

Consistent with the mediation findings summarized in Table 5, the Impulse-Related Energy Transfer Efficiency factor was significantly associated with CHS (B=0.62, SE=0.15, p<0.001). However, Trunk Sequencing at Impact ($M_{1}$) did not independently predict CHS once $M_{2}$ was incorporated into the model (B=0.17, SE=0.13, p=0.19). This indicates that the influence of segmental sequencing is largely accounted for by the efficiency of mechanical impulse transmission. The final mediation model, which controlled for skill group as a significant covariate (β=0.71, p<0.001), explained 55% of the total variance in clubhead speed ($R^{2}=.55$). Despite the small sample size (N=30), a post hoc power analysis based on the observed effect size ($f^{2}=0.35$) confirmed that the achieved power for the indirect effect via Impulse-Related Energy Transfer Efficiency exceeded 0.80, meeting the requirements for reliable effect detection.

This coceptual diagram (Figure 5) illustrates the sequential relationships and hypothesized statistical pathways between foot pressure/center of pressure dynamics, segmental sequencing, impulse-based energy transfer, and clubhead speed. It should be noted that this diagram represents a statistical model derived from cross-sectional mediation analysis rather than a confirmed longitudinal causal pathway. The model highlights how these associations are moderated by skill level (14, 20, 46–48). Key mediators such as efficient foot dynamics (12, 48) are associated with optimal segmental sequencing (4, 6, 49), which in turn is related to effective impulse-based energy transfer (34, 48, 50) to maximize clubhead speed.

#### 3.5.4 Summary of key findings

The principal results of this study can be summarized as follows:

- Early-phase foot pressure and COP displacement did not directly predict clubhead speed.
- Impulse-related energy transfer efficiency demonstrated a significant and robust association with clubhead speed.
- Trunk sequencing differentiated skill level but did not independently predict clubhead speed once impulse-based energy transfer was accounted for.

## 4 Discussion

The present study offers evidence for a mechanistic explanation of how lower-limb mechanics are associated with clubhead speed during the golf swing by highlighting the significant role of impulse-based energy transfer along the kinetic chain. By integrating foot pressure distribution, COP dynamics, segmental sequencing, and joint-level energy flow within a unified mediation framework, these findings extend existing golf biomechanics research beyond isolated kinematic or kinetic descriptors. Notably, the results provide a potential explanation for why previous studies have reported inconsistent relationships between foot–ground interaction variables and CHS, suggesting a hierarchical organization of golf swing performance.

### 4.1 Principal findings

The principal finding of this study is that CHS is not directly associated with early-phase foot pressure or COP displacement but is instead consistent with the efficiency of impulse-based energy transfer during the late backswing–to–impact transition. Although COP and foot pressure variables were associated with trunk sequencing and intermediate energy-flow constructs, their direct effects on CHS became non-significant once mediating mechanisms were included.

Serial mediation analysis identified impulse-related energy transfer efficiency as a significant mechanical pathway associated with CHS. The indirect effect of early-phase COP displacement on CHS through impulse-based energy transfer efficiency was statistically significant (95% CI [0.07, 0.63]), supporting the proposed energy-based transmission mechanism. This suggests that early COP behavior facilitates more effective impulse transmission from proximal to distal segments, rather than exerting a direct mechanical influence on the club head.

In contrast, Trunk Sequencing at Impact, while strongly differentiating between professional and amateur golfers, did not independently predict CHS once energy transfer efficiency was accounted for. This pattern suggests that kinematic sequencing alone may be insufficient to explain performance outcomes without considering how effectively mechanical energy is transmitted through the kinetic chain.

Collectively, these findings support a hierarchical organization of the kinetic chain, in which force generation at the lower extremities, coordination of segmental motion, and efficient energy transmission represent distinct yet interdependent contributors to clubhead speed. From a biomechanical perspective, impulse-based energy flow appears to be a significant contributor to performance, offering a mechanistically informative target for assessment and training. This superior system-level organization in professional golfers can be operationalized as a more efficient kinetic chain characterized by reduced mechanical energy leakage, optimized intersegmental timing, and highly adaptive motor variability.

### 4.2 Foot pressure and COP as mechanical inputs rather than performance outputs

Previous golf biomechanics research has frequently examined foot pressure distribution and COP displacement as potential predictors of swing performance. While these variables are widely recognized as foundational for establishing a stable base and generating ground reaction forces, the present findings suggest that their role may be best understood as foundational mechanical inputs or boundary conditions, rather than as direct predictors of CHS.

The absence of a statistically significant direct effect of early COP variables on CHS, after accounting for mediating factors, appears consistent with classical biomechanical theory, which posits that ground reaction forces contribute to performance only to the extent that they are effectively transmitted through the musculoskeletal system (4, 23). In this context, COP behavior initiates force production but may not necessarily lead to effective energy delivery to the club unless subsequent intersegmental transfer mechanisms are optimized.

This perspective provides a potential framework for understanding inconsistencies in the literature, where some studies report strong associations between COP metrics and CHS (48), while others observe weak or inconsistent relationships (46, 47, 50). The present results suggest that such discrepancies may arise from failing to account for downstream mediators, particularly impulse-based energy transfer across joints.

### 4.3 Impulse-based energy transfer as the central performance mechanism

A notable contribution of this study lies in identifying impulse-based energy transfer as a significant mechanical contributor to clubhead speed. Unlike instantaneous kinematic variables, impulse captures both the magnitude of force and its temporal characteristics, making it particularly relevant for explosive, high-speed actions such as the golf swing (34), throwing, or striking.

The significant mediating role of impulse-based energy transfer suggests that performance appears to be less associated with the absolute amount of force generated early in the swing and more aligned with how effectively that force is transmitted across segments. This finding supports and extends core-to-extremity sequencing theory (6, 34, 51), emphasizing that effective sequencing must culminate in uninterrupted energy flow toward the terminal segments (4, 48, 52).

Notably, the indirect pathway from COP to CHS, achieved through trunk sequencing alone, was not significant, whereas the pathway involving impulse-based energy transfer remained robust. This suggests that optimal timing without effective force transmission is insufficient, reinforcing the notion that sequencing should be evaluated in conjunction with kinetic efficiency, rather than as an isolated temporal phenomenon.

### 4.4 Trunk sequencing: skill differentiation vs. performance determination

Trunk sequencing has frequently been highlighted as a significant factor associated with golf performance, with elite golfers often exhibiting earlier and more pronounced trunk rotation patterns (53). In the present study, trunk sequencing distinguished professional and amateur golfers, further supporting its role as a potential indicator of technical proficiency and a coordinative factor within the kinetic chain.

However, a crucial distinction emerges that trunk sequencing did not independently predict CHS once impulse-based mediators were included in the model. The moderate-to-strong correlation (r = 0.63) between trunk sequencing and impulse-based energy transfer indicates that while these constructs are related, they represent distinct biomechanical phenomena. Segmental sequencing serves as a kinematic framework, describing the optimal timing and temporal ordering of peak segmental velocities. However, sequencing alone does not account for the magnitude of mechanical work or the efficiency of its transmission. Energy-flow and impulse-based metrics provide greater explanatory depth by quantifying the actual kinetic energy successfully transferred across the kinetic chain. This suggests that while proper timing (sequencing) is a prerequisite for an effective swing, the primary driver of clubhead speed is the efficiency with which mechanical impulse is generated and transmitted, which ultimately dictates performance more directly than temporal coordination alone. This suggests that trunk sequencing functions primarily as a facilitator of movement timing, enabling effective energy transfer, rather than as a direct generator of clubhead speed.

While idealized kinematic sequencing is often considered optimal for efficient power transfer, research indicates that movement variability is a hallmark of skilled golf performance (20, 54). In the pursuit of maximal clubhead speed, elite golfers employ functional adaptations in their force-generation strategies, which may involve strategic adjustments to the conventionally defined “ideal” kinematic sequence. This purposeful integration of kinematic precision and explosive force application, supported by the functional variability observed in skilled movements, (46, 54), suggests that elite golfers prioritize the optimization of the impulse-based energy transfer mechanism over rigid adherence to a singular movement template. Accordingly, suggests that efficient trunk motion facilitates impulse transmission; however, its isolated contribution to CHS remains limited without sufficient intersegmental energy transfer.

### 4.5 Group effects as system-level organization

The observed group differences in clubhead speed, trunk sequencing, and impulse-related energy transfer efficiency suggest that professional and amateur golfers exhibit distinct patterns in their system-level organization of the golf swing (4, 48). Professional golfers demonstrated a higher capacity to integrate force generation, coordination, and energy transmission into a coherent kinetic strategy that minimizes energy leakage and maximizes distal output.

Importantly, as shown in Table 4, the “Group” variable remained the strongest and most significant predictor of CHS (β=0.71,p<0.001) even after accounting for all biomechanical mediators. This indicates a state of “partial mediation,” suggesting that while trunk sequencing and energy transfer efficiency are vital components of the kinetic chain, they do not fully capture the entire spectrum of professional expertise. Other unmeasured factors inherent to elite status—such as greater explosive muscular power, superior grip-to-clubhead dynamics, and more refined neuromuscular control—likely account for the remaining variance in performance.

This integrated system-level organization is associated with more consistent proximal-to-distal sequencing (8, 55), optimized intersegmental energy transfer (4, 6), and observed motor control and adaptability (25). Professional golfers in this study demonstrated balance and stability profiles that may facilitate effective weight transfer and targeted force application while being associated with reduced extraneous movements (56). Moreover, skilled golfers exhibited a tendency to regulate movement variability across swing phases, showing increased variability during power generation and reduced variability near impact (57).

Collectively, these characteristics suggest that skill-related performance differences in golf may be attributed not merely to greater strength or speed, but potentially to a more integrated and efficient coordination of the kinetic chain.

### 4.6 Theoretical implications

The findings of this study contribute to golf biomechanics by shifting analytical focus from isolated predictors to meditating mechanical pathways. By suggesting that impulse-based energy transfer mediates the relationship between lower-limb mechanics and clubhead speed, the results are consistent with a hierarchical kinetic chain model in which:

- Foot pressure and COP establish mechanical boundary conditions for force generation.
- Segmental sequencing organizes movement timing and coordination.
- Impulse-based energy transfer serves as a significant predictor of performance output through efficient intersegmental transmission.

This framework may help explain divergent findings in the literature and provides a robust template for examining complex multi-joint athletic movements that require coordinated kinetic and kinematic interactions (47).

### 4.7 Practical implications

From a practical standpoint, these findings suggest that training interventions may benefit from emphasizing intersegmental impulse-based energy transfer, alongside existing focuses on ground reaction forces and COP trajectories. Coaching strategies that emphasize force magnitude without addressing transmission efficiency could potentially result in suboptimal performance gains.

The results also offer potential implications for footwear and insole design. Rather than maximizing localized pressure, equipment could be developed ro support stable force transmission pathways that facilitate effective impulse flow through the kinetic chain, aligning with emerging sensor-based approaches to performance monitoring and technique optimization (12, 58).

### 4.8 Limitations and future directions

Several limitations should be acknowledged. Although the participant group was relatively homogeneous, a larger and more diverse sample could potentially facilitate the examination of sex-based differences and further validation of the identified mediation pathways. Specifically, future research should investigate the applicability of these findings to female golfers, older populations, and elite professionals with handicaps below 5, who may exhibit distinct biomechanical compensation strategies. The use of maximal-effort swings, while appropriate for isolating mechanisms related to clubhead speed, might not fully represent performance variability during typical 7-iron play. Furthermore, these results, obtained in a controlled laboratory setting, may have limited ecological validity as they might not fully replicate on-course performance under competitive pressure.

Additionally, incorporating electromyography or more detailed joint power analyses could further elucidate the neuromuscular mechanisms underlying impulse transfer efficiency. Furthermore, the 20 Hz sampling frequency of the plantar pressure system, while sufficient for the phase-integrated and impulse-based analysis central to this study, represents a lower temporal resolution compared to the kinematic and force platform data. This resolution may limit the capture of high-frequency, instantaneous pressure transients or rapid center-of-pressure shifts during the most dynamic moments of the transition and impact. Specifically, a 20 Hz sampling frequency may miss rapid, transient pressure shifts occurring during the impact interval; thus, future work utilizing higher-frequency insole systems (e.g., >100 Hz) is suggested.

Furthermore, the sample size (N=30), relative to the number of variables in each PCA block, may influence the stability of the factor loadings. Although PCA served as an effective tool for dimensionality reduction, the results should be interpreted with caution. Similarly, while the final regression model explained a significant proportion of the variance in clubhead speed ($R^{2}=0.55$, Adjusted $R^{2}=0.52$), the potential for overfitting in a relatively small sample warrants acknowledgment. To mitigate this risk, we prioritized Adjusted $R^{2}$ as a more conservative estimate and utilized a 5,000-resample bias-corrected bootstrap procedure, which confirmed that the estimated coefficients remained stable.

Regarding the mediation analysis, while detecting indirect effects typically requires larger samples, the primary indirect effect through energy transfer efficiency ($M_{2}$) demonstrated a robust effect size (B = 0.31, 95% CI [0.07, 0.63]). A post-hoc power analysis indicated that the achieved power for this specific pathway exceeded 0.80, suggesting the current sample size was sufficient to detect this primary mechanistic link. Finally, because these data are cross-sectional, results should be interpreted as being statistically consistent with the proposed hierarchical organization rather than as direct evidence of causality. Therefore, longitudinal designs are necessary to definitively establish causal relationships. Future research could also benefit from integrating interpretable machine learning frameworks applied to simulation-derived biomechanical features to handle high-dimensional data more effectively

### 4.9 Integrative mechanistic synthesis

Taken together, the present findings are consistent with hierarchical organization of the golf swing kinetic chain in which lower-limb force generation, segmental coordination, and impulse-based energy transmission represent distinct yet interdependent contributors to clubhead speed. Early-phase foot pressure and COP behavior were not observed to have a significant direct association with CHS; instead, their contribution appears to be realized through facilitating impulse transmission during the transition from the late backswing to impact.

Within this framework, impulse-based energy transfer efficiency emerges as a significant factor associated with performance. While COP-related variables and trunk sequencing influence intermediate biomechanical constructs, their direct effects on clubhead speed are attenuated once energy transfer efficiency is considered. These findings highlight the potential importance of evaluating kinematic variables in conjunction with kinetic efficiency and suggest the value of energy-based metrics for understanding golf swing performance.

## 5 Conclusions

This study presents a mechanistic framework suggesting that clubhead speed during the golf swing is closely associated with the efficiency of impulse-based energy transfer across segments, rather than solely by the direct effects of early-phase foot pressure or center-of-pressure behavior. By integrating plantar pressure distribution, COP dynamics, segmental sequencing, and joint-level energy flow within a unified analytical approach, the findings provide a novel perspective on how lower-limb mechanics dictate golf swing performance.

The results indicate that although effective weight transfer and plantar pressure modulation contribute to impulse generation, their influence on CHS is predominantly indirect. Serial mediation analyses reveal that a significant pathway linking foot–ground interaction to CHS operates through impulse-based energy transfer efficiency during the late backswing-to-impact transition. While trunk sequencing differentiates skill levels and serves as an important coordinative facilitator, its direct mediating role was not statistically significant once energy transfer efficiency was considered, suggesting that effective timing alone may be insufficient without efficient kinetic transmission.

Comparisons between professional and high-level amateur golfers further suggest that professional performance is associated with a more integrated system-level organization of the kinetic chain. Professional golfers demonstrated a higher capacity to integrate force generation, coordination, and energy transmission, resulting in the more effective utilization of lower-limb–generated impulse rather than merely greater isolated force production.

In summary, this study contributes to golf swing biomechanics by delineating a potential mechanistic pathway from foot–ground interaction to clubhead speed mediated by impulse-based energy transfer. This hierarchical perspective provides a refined foundation for evidence-based coaching and technology-assisted performance evaluation. To advance these findings, high-priority next steps should include intervention studies to determine if targeted training of impulse-based energy transfer directly improves CHS; the development of real-time, sensor-based feedback tools for on-course performance monitoring; and investigating the applicability of this framework to other high-velocity rotational sports.

## Data Availability

The raw data supporting the conclusions of this article will be made available by the authors, without undue reservation.

## Footnotes

^1^Qualisys AB. *Qualisys sports marker set reference guide*. Gothenburg: Qualisys AB (2023). Available at: https://www.qualisys.com/software/qualisys-track-manager/.
