# Supplementary material for: Foot–ground interaction and clubhead speed: impulse-based energy transfer as the key mechanism in the golf swing
Source: Front Sports Act Living. 2026 Apr 13;8:1790645. doi: 10.3389/fspor.2026.1790645 (PMC13111475; doi:10.3389/fspor.2026.1790645)
Supplement: Supplementary file 1 [file Datasheet1.docx]

**Supplemental Figure S1:**

**Panel A: Foot Pressure Distribution**


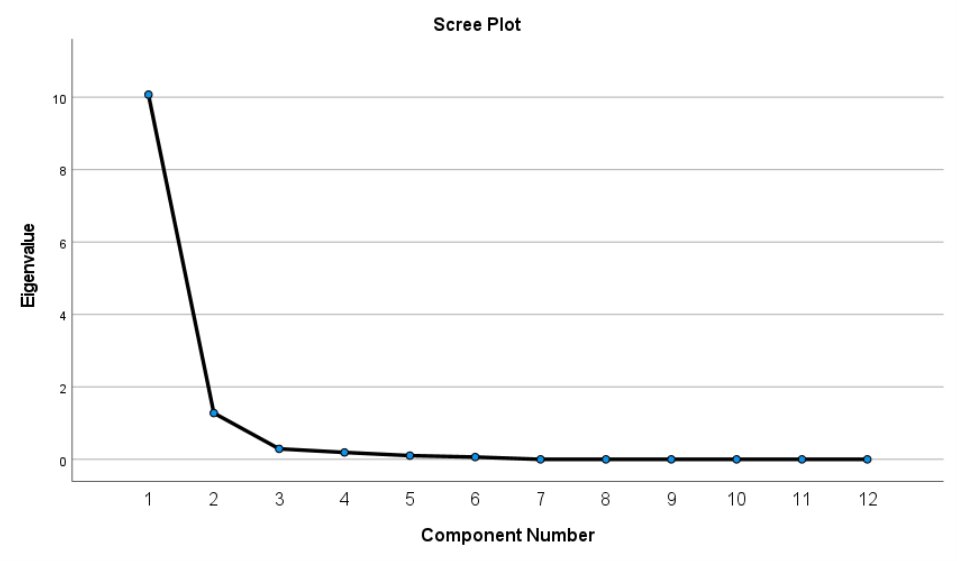


**Panel B: Center-of-Pressure Dynamics**


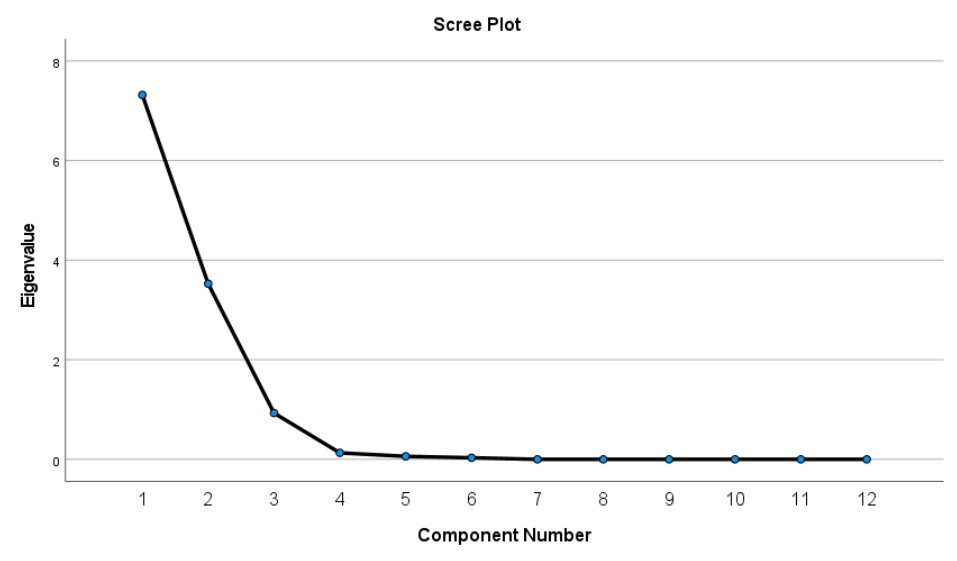


**Panel C: Trunk Kinematic Sequencing**


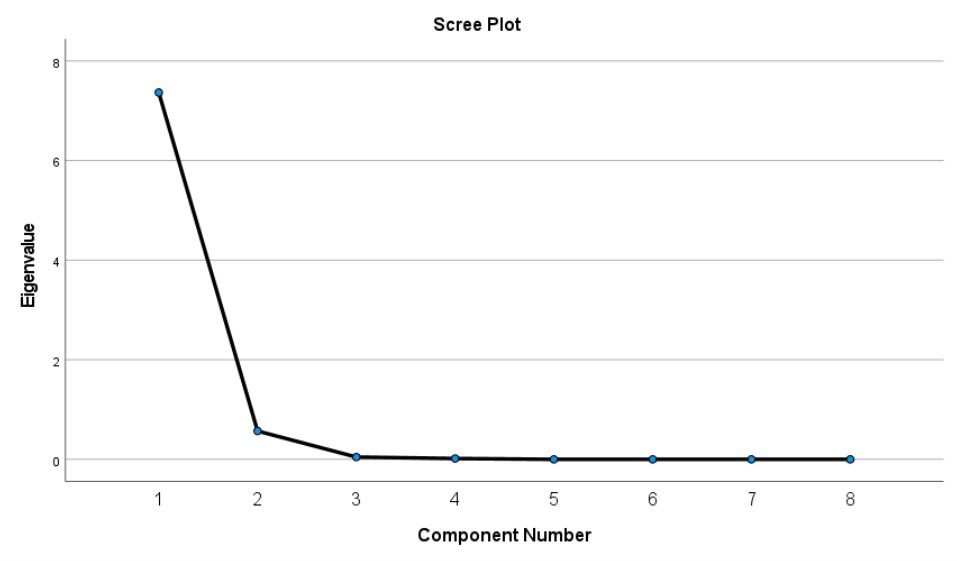


**Panel D: Impulse-Based Energy Transfer Efficieny**


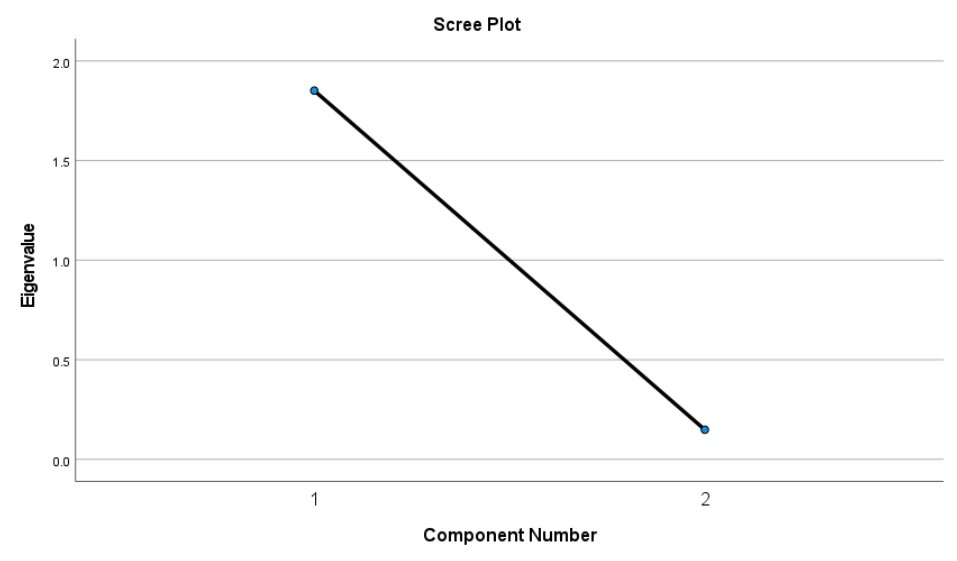


Supplemental Figure S1. Representative scree plots demonstrate eigenvalue distributions for the four primary biomechanical blocks: (A) Foot pressure distribution, (B) Center-of-pressure dynamics, (C) Trunk kinematic sequencing, and (D) Impulse-based energy transfer efficiency. In each panel, the horizontal dashed line indicates the Kaiser criterion (eigenvalue = 1.0). The selection of factors for subsequent analyses was consistently based on the "elbow" of the plot and the Kaiser criterion across all analyzed swing phases, ensuring robust dimensionality reduction while preserving essential variance.

**Supplemental Figure S2:**

Statistical diagram of the serial mediation model illustrating the pathways between Foot–Ground Interaction and Clubhead Speed.


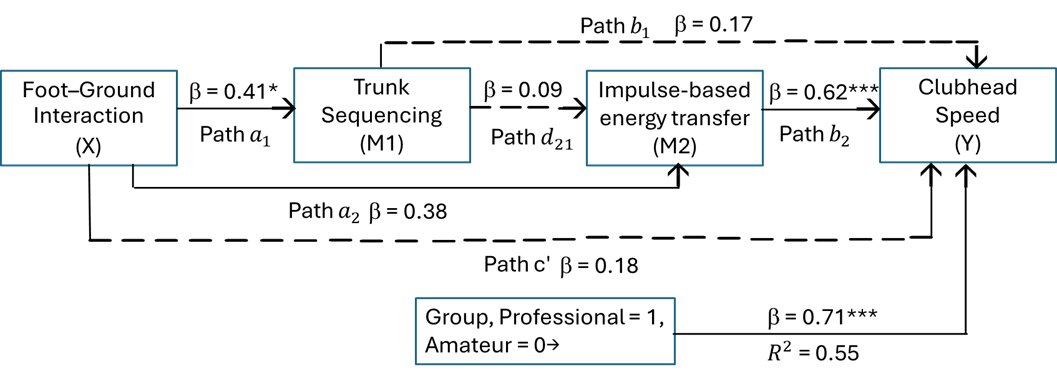


Statistical diagram of the serial mediation model illustrating the mechanistic pathways from early-phase foot pressure dynamics to clubhead speed. The model includes Early-Phase COP Displacement (X), Trunk Sequencing at Impact (M1), Impulse-Related Energy Transfer Efficiency (M2), and Clubhead Speed (Y). Values presented on the paths are unstandardized regression coefficients (B). Solid lines represent statistically significant paths, whereas dashed lines indicate non-significant relationships. Significant paths were observed from X to M1 (a₁ = 0.41*) and from X to M2 (a₂ = 0.38*). Impulse-related energy transfer efficiency (M2) significantly predicted clubhead speed (b₂ = 0.62***), whereas trunk sequencing (M1) was not a significant predictor (b₁ = 0.17, ns). The direct effect of X on Y was non-significant (c′ = 0.21, ns), and the sequential pathway between mediators was also non-significant (d₂₁ = 0.09, ns). Skill group was included as a covariate predicting clubhead speed (β = 0.71, p < .001). The model explained 55% of the variance in clubhead speed (R² = .55).

Note. Indirect effects were estimated using 5,000 bootstrap resamples. Detailed coefficients, standard errors, and 95% confidence intervals for all direct and indirect effects are reported in Tables 4 and 5. p < .05, p < .01, p < .001; ns = non-significant.
